# Correction: Problem-solving interventions and depression among adolescents and young adults: A systematic review of the effectiveness of problem-solving interventions in preventing or treating depression

**DOI:** 10.1371/journal.pone.0315129

**Published:** 2024-12-03

**Authors:** Kristina Metz, Jane Lewis, Jade Mitchell, Sangita Chakraborty, Bryce D. McLeod, Ludvig Bjørndal, Robyn Mildon, Aron Shlonsky

There are errors in the Author Contributions. The correct contributions are:

**Conceptualization:** Kristina Metz, Jane Lewis, Bryce D. McLeod, Robyn Mildon, Aron Shlonsky

**Data curation:** Kristina Metz, Jade Mitchell, Sangita Chakraborty, Ludvig Bjørndal

**Formal analysis:** Kristina Metz, Aron Shlonsky

**Methodology:** Aron Shlonsky

**Project administration:** Robyn Mildon

**Writing–original draft:** Kristina Metz, Jane Lewis, Aron Shlonsky

**Writing–review & editing:** Kristina Metz, Bryce D. McLeod, Robyn Mildon, Aron Shlonsky.
