# Supplementary material for: Problem-solving interventions and depression among adolescents and young adults: A systematic review of the effectiveness of problem-solving interventions in preventing or treating depression
Source: PLoS One. 2023 Aug 29;18(8):e0285949. doi: 10.1371/journal.pone.0285949 (PMC10464969; doi:10.1371/journal.pone.0285949)
Supplement: S1 File — (PDF) [file pone.0285949.s001.pdf]

S1 File. List of excluded studies

1. Abas M, Nyamayaro P, Bere T, Saruchera E, Mothobi N, Simms V, et al. Feasibility and acceptability of a task-shifted intervention to enhance adherence to HIV medication and improve depression in people living with HIV in Zimbabwe, a low income country in sub-Saharan Africa. AIDS and Behavior. 2018; 22(1), 86-101. doi: 10.1007/s10461-016-1659-4
2. Arends I, Almansa J, Stansfeld S, Amick B, van der Klink J, Bultmann U. One-year trajectories of mental health and work outcomes post return to work in patients with common mental disorders. Journal of Affective Disorders. 2019; 257, 263-270. doi: 10.1016/j.jad.2019.07.018
3. Asnani MR, Francis D, Knight-Madden J, Chang-Lopez S, King L, Walker S. Integrating a problem-solving intervention with routine care to improve psychosocial functioning among mothers of children with sickle cell disease: A randomized controlled trial. PLoS ONE. 2021; 16(6): e0252513. doi: 10.1371/journal.pone.0252513
4. Auslander W, McGinnis H, Tlapek S, Smith P, Foster A, Edmond T, et al. Adaptation and implementation of a trauma-focused cognitive behavioral intervention for girls in child welfare. American Journal of Orthopsychiatry. 2017; 87(3), 206-215. doi: 10.1037/ort0000233
5. Auslander W, Sterzing P, Threlfall J, Gerke D, Edmond T. Childhood abuse and aggression in adolescent girls involved in child welfare: The role of depression and posttraumatic stress. Journal of Child & Adolescent Trauma. 2016; 9(4), 359-368. doi: 10.1007/s40653-016-0090-3

6. Balck F, Zschieschang A, Zimmermann A, Ordemann R. A randomized controlled trial of problem-solving training (PST) for hematopoietic stem cell transplant (HSCT) patients: Effects on anxiety, depression, distress, coping and pain. Journal of Psychosocial Oncology. 2019; 37(5), 541-556. doi: 10.1080/07347332.2019.1624673

7. Barrera M, Atenafu E, Nathan PC, Schulte F, Hancock K. Depression and quality of life in siblings of children with cancer after group intervention participation: A randomized control trial. Journal of Pediatric Psychology. 2018; 43(10), 1093-1103. doi: 10.1093/jpepsy/jsy040

8. Barrett JE, Williams JW, Oxma TE, Frank E, Katon W, Sullivan M, et al. Treatment of dysthymia and minor depression in primary care: a randomized trial in patients aged 18 to 59 years. Journal of Family Practice. 2001; 50(5), 405-412. doi: 10.1001/jama.284.12.1519

9. Becker-Weidman EG, Jacobs RH, Reinecke MA, Silva SG, March JS. Social problem-solving among adolescents treated for depression. Behaviour Research and Therapy. 2010; 48(1), 11-18. doi: 10.1016/j.brat.2009.08.006

10. Bedford LA, Dietch JR, Taylor DJ, Boals A, Zayfert C. Computer-guided problem-solving treatment for depression, PTSD, and insomnia symptoms in student veterans: A pilot randomized controlled trial. Behavior Therapy. 2018; 49(5), 756-767. doi: 10.1016/j.beth.2017.11.010

11. Bell AC, D'Zurilla TJ. Problem-solving therapy for depression: A meta-analysis. Clinical Psychology Review. 2009; 29(4), 348-353. doi: 10.1016/j.cpr.2009.02.003

12. Bentacourt, T. Adapting mental health interventions for war-affected youth through employment programs. 2018. ClinicalTrials.gov Identifier: NCT02561949 Retrieved from: https://clinicaltrials.gov/show/NCT02561949
13. Bentacourt, T. Trial of the Youth readiness intervention. 2018. ClinicalTrials.gov Identifier: NCT01684488. Retrieved from: https://clinicaltrials.gov/show/NCT01684488
14. Bentacourt, T. Sub-trial of the Youth Readiness Intervention (YRI): Treatment of control group and addition of stress biomarkers. 2019. ClinicalTrials.gov Identifier: NCT02128568. Retrieved from: https://clinicaltrials.gov/show/NCT02128568.
15. Betancourt TS, McBain R, Newnham EA, Akinsulure-Smith AM, Brennan RT, Weisz JR, et al. A behavioral intervention for war-affected youth in Sierra Leone: A randomized controlled trial. Journal of the American Academy of Child & Adolescent Psychiatry. 2014; 53(12), 1288-1297. doi: 10.1016/j.jaac.2014.09.011
16. Boele FW, Klein M, Verdonck-de Leeuw IM, Cuijpers P, Heimans JJ, Snijders T, et al. Internet-based guided self-help for glioma patients with depressive symptoms: a randomized controlled trial. Journal of Neuro-oncology. 2018; 137(1), 191-203. doi: 10.1007/s11060-017-2712-5
17. Bolton P, Bass J, Neugebauer R, Verdeli H, Clougherty KF, Wickramaratne P et al. Group interpersonal psychotherapy for depression in rural Uganda: A randomized controlled trial. JAMA. 2003; 289(23), 3117-3124. doi: 10.1001/jama.289.23.3117

18. Bombardier CH, Bell KR, Temkin NR, Fann JR, Hoffman J, Dikmen S. The efficacy of a scheduled telephone intervention for ameliorating depressive symptoms during the first year after traumatic brain injury. The Journal of Head Trauma Rehabilitation. 2009; 24(4), 230-238. doi: 10.1097/HTR.0b013e3181ad65f0

19. Boston Medical Centre. Improving preterm outcomes by safeguarding maternal mental health. 2018. ClinicalTrials.gov Identifier: NCT01892982. Retrieved from: https://clinicaltrials.gov/show/NCT01892982

20. Buntrock C, Ebert DD, Lehr D, Cuijpers P, Riper H, Smit F, et al. Evaluating the efficacy and cost-effectiveness of web-based indicated prevention of major depression: Design of a randomised controlled trial. BMC Psychiatry. 2014; 14(25). doi: 10.1186/1471-244X-14-25

21. Burckhardt R, Manicavasagar V, Batterham PJ, Hadzi-Pavlovic D. A randomized controlled trial of strong minds: A school-based mental health program combining acceptance and commitment therapy and positive psychology. Journal of School Psychology. 2016; 57, 41-52. doi: 10.1016/j.jsp.2016.05.008

22. Cardemil EV. The prevention of depressive symptoms in inner-city, minority middle school students. Dissertation Abstracts International: Section B: The Sciences and Engineering. 2000; 61(3-B), 1627. doi: 10.1016/j.brat.2006.03.010

23. Cardemil E, Reivich KJ, Beevers CG, Seligman ME, James J. The prevention of depressive symptoms in low-income, minority children: Two-year follow-up. Behaviour Research and Therapy. 2007; 45*(*2), 313-327. doi: 10.1037/1522-3736.5.1.58a

24. Cardemil EV, Reivich KJ, Seligman ME. The prevention of depressive symptoms in low-income minority middle school students. Prevention & Treatment. 2002; 5(1). doi:10.1037/1522-3736.5.1.58a

25. Carey JC, Wade SL, Wolfe CR. Lessons learned: the effect of prior technology use on Web-based interventions. Cyberpsychology & Behavior. 2008; 11(2), 188-195. doi: 10.1089/cpb.2007.0025

26. Chibanda D, Weiss HA, Verhey R, Simms V, Munjoma R, Rusakaniko S. Effect of a primary care-based psychological intervention on symptoms of common mental disorders in Zimbabwe: A randomized clinical trial. JAMA, 2016; 316(24), 2618-2626. doi: 10.1001/jama.2016.19102

27. Chu, B. Transdiagnostic Individual Behavioral Activation and Exposure Therapy. 2019. ClinicalTrials.gov Identifier: NCT03412227. Retrieved from: https://clinicaltrials.gov/show/NCT03412227

28. Ciechanowski PS, Russo JE, Katon WJ, Von Korff M, Simon GE, Lin EH, et al. The association of patient relationship style and outcomes in collaborative care treatment for depression in patients with diabetes. Medical Care. 2006; 44(3), 283-291. doi: 10.1097/01.mlr.0000199695.03840.0d

29. Dalgleish T, Bevan A, McKinnon A, Breakwell L, Mueller V, Chadwick I, et al. A comparison of MEmory Specificity Training (MEST) to education and support (ES) in the treatment of recurrent depression: Study protocol for a cluster randomised controlled trial. Trials. 2014; 15(293). doi: 10.1186/1745-6215-15-293

30. DaWalt LS, Greenberg JS, Mailick MR. Transitioning together: A multi-family group psychoeducation program for adolescents with ASD and their parents. Journal of Autism and Developmental Disorders. 2018; 48(1), 251-263. doi: 10.1007/s10803-017-3307-x

31. Dawson K, Joscelyne A, Meijer C, Steel Z, Silove D, Bryant RA. A controlled trial of trauma-focused therapy versus problem-solving in Islamic children affected by civil conflict and disaster in Aceh, Indonesia. Australian and New Zealand Journal of Psychiatry. 2018; 52(3), 253-261. doi: 10.1177/0004867417714333

32. De Cuyper S, Timbremont B, Braet C, De Backer V, Wullaert T. Treating depressive symptoms in schoolchildren: A pilot study. European Child & Adolescent Psychiatry. 2004; 13(2), 105-114. doi: 10.1007/s00787-004-0366-2

33. Diamond GS, Levy S. Bindungsorientierte Familientherapie als ambulante Nachsorge für Jugendliche nach Suizidversuch. PiD-Psychotherapie im Dialog. 2012; 13(02), 41-45. doi: /10.1055/s-0032-1304976

34. Donaldson C, Lam D. Rumination, mood and social problem-solving in major depression. Psychological Medicine. 2004; 34(7), 1309-1318. doi: 10.1017/S0033291704001904

35. Dour HJ, Chorpita BF, Lee S, Weisz JR; Research Network on Youth Mental Health. Sudden gains as a long-term predictor of treatment improvement among children in community mental health organizations. Behaviour Research and Therapy. 2013; 51(9), 564-572. doi: 10.1016/j.brat.2013.05.012

36. Dowrick C, Dunn G, Ayuso-Mateos JL, Dalgard OS, Page H, Lehtinen V, et al. Problem solving treatment and group psychoeducation for depression:

Multicentre randomised controlled trial. British Medical Journal. 2000; 321(7274), 1450. doi: 10.1136/bmj.321.7274.1450

37. Eskin M, Ertekin K, Demir H. Efficacy of a problem-solving therapy for depression and suicide potential in adolescents and young adults. Cognitive Therapy and Research. 2008; 32(2), 227–245. doi: 10.1007/s10608-007-9172-8

38. Espinosa D. An Internet-based Program for Prevention and Early Intervention of Adolescent Depression. 2018. ClinicalTrials.gov Identifier: NCT02780232. Retrieved from: https://clinicaltrials.gov/show/NCT02780232

39. Espinosa D. Pilot study of an internet-based program for prevention and early intervention of adolescent depression. 2018. ClinicalTrials.gov Identifier: NCT03047512. Retrieved from: https://clinicaltrials.gov/show/NCT03047512

40. Fatima B, Cohen N. Participatory intervention to reduce maternal depression and under five child morbidity. 2017. ClinicalTrials.gov Identifier: NCT02047357. Retrieved from: https://clinicaltrials.gov/show/NCT02047357

41. Feinberg E. Supporting the well being of families of young children with autism spectrum disorders. 2013. ClinicalTrials.gov Identifier: NCT01021384. Retrieved from: https://clinicaltrials.gov/show/NCT01021384

42. Feinberg E. Reducing risk after an adverse pregnancy outcome. 2014. ClinicalTrials.gov Identifier: NCT01182363. Retrieved from: https://clinicaltrials.gov/show/NCT01182363

43. Feinberg E, Augustyn M, Fitzgerald E, Sandler J, Ferreira-Cesar Suarez Z, Chen N, et al. Improving maternal mental health after a child's diagnosis of autism

spectrum disorder: Results from a randomized clinical trial. JAMA Pediatric. 2014; 168(1), 40-46. doi: 10.1001/jamapediatrics.2013.3445

44. Frank E, Rucci P, Katon W, Barrett J, Williams JW, Oxman T, et al. Correlates of remission in primary care patients treated for minor depression. General Hospital Psychiatry. 2002; 24(1), 12-19. doi: 10.1016/S0163-8343%2801%2900173-6

45. Gaete J, Martinez V, Fritsch R, Rojas G, Montgomery AA, Araya R. Indicated school-based intervention to improve depressive symptoms among at risk Chilean adolescents: A randomized controlled trial. BMC Psychiatry. 2016; 16(276). doi: 10.1186/s12888-016-0985-4

46. Gerkensmeyer JE, Johnson CS, Scott EL, Oruche UM, Lindsey LM, Austin J, et al. Problem-solving intervention for caregivers of children with mental health problems. Archives of Psychiatric Nursing. 2013; 27(3), 112-120. doi: 10.1016/j.apnu.2013.01.004

47. Gillham JE. R4Power: An Online Resilience Program for Adolescents (R4Power). 2008. ClinicalTrials.gov Identifier: NCT00641368. Retrieved from: https://clinicaltrials.gov/ct2/show/NCT00641368

48. Gillham JE, Reivich KJ, Brunwasser SM, Freres DR, Chajon ND, Kash-MacDonald VM. Evaluation of a group cognitive-behavioral depression prevention program for young adolescents: A randomized effectiveness trial. Journal of Clinical Child & Adolescent Psychology. 2012; 41(5), 621-639. doi: 10.1080/15374416.2012.706517

49. Guild PA, Freeman VA, Shanahan E. Promising Practices to Prevent Adolescent Suicide: What We Can Learn From New Jersey. Cecil G. Sheps Center. 2004.

https://www.academia.edu/download/49263906/Promising_Practices_to_Prevent_Adolescen20161001-31060-10x0ssc.pdf

50. Gureje O, Oladeji BD, Araya R, Montgomery AA, Kola L, Kirmaye L, et al. Expanding care for perinatal women with depression (EXPONATE): Study protocol for a randomized controlled trial of an intervention package for perinatal depression in primary care. BMC Psychiatry. 2015; 15(136), 1-9. doi: 10.1186/s12888-015-0537-3

51. Gussak D. The effects of art therapy on male and female inmates: Advancing the research base. The Arts in Psychotherapy. 2009; 36(1), 5-12. doi: 10.1016/j.aip.2008.10.002

52. Hallford DJ, Mellor D. Cognitive-reminiscence therapy and usual care for depression in young adults: Study protocol for a randomized controlled trial. Trials. 2013; 14(343). doi:10.1186/1745-6215-14-343

53. Hameed M, O'Doherty L, Gilchrist G, Tirado-Muñoz J, Taft A, Chondros P, et al. Psychological therapies for women who experience intimate partner violence. Cochrane Database of Systematic Reviews. 2020; (7). doi: 10.1002%2F14651858.CD013017.pub2

54. Harley R, Petersen T, Scalia M, Papakostas GI, Farabaugh A, Fava M. Problem-solving ability and comorbid personality disorders in depressed outpatients. Depression and Anxiety. 2006; 23(8), 496-501. doi: 10.1002/da.20194

55. Hatcher S, Sharon C, Parag V, Collins N. Problem-solving therapy for people who present to hospital with self-harm: Zelen randomised controlled trial. The

British Journal of Psychiatry. 2011; 199(2), 310-316. doi: 10.1192/bjp.bp.110.090126

56. Hedemann ER, Frazier SL. Leveraging after-school programs to minimize risks for internalizing symptoms among urban youth: Weaving together music education and social development. Administration and Policy in Mental Health and Mental Health Services Research. 2017; 44(5), 756-770. doi: 10.1007/s10488-016-0758-x

57. Heller HM, Hoogendoorn AW, Honig A, Broekman BF, van Straten A. The effectiveness of a guided internet-based tool for the treatment of depression and anxiety in pregnancy (MamaKits Online): Randomized controlled trial. Journal of Medical Internet Research. 2020; 22(3), e15172. doi: 10.2196/15172

58. Hetrick SE, Yuen HP, Bailey E, Cox GR, Templer K, Rice, SM, et al. Internet-based cognitive behavioural therapy for young people with suicide-related behaviour (Reframe-IT): A randomised controlled trial. Evidence-Based Mental Health. 2017; 20(3), 76-82. doi: 10.1136/eb-2017-102719

59. Hoek W, Schuurmans J, Koot HM, Cuijpers P. Prevention of depression and anxiety in adolescents: A randomized controlled trial testing the efficacy and mechanisms of Internet-based self-help problem-solving therapy. Trials. 2019; 10(93). doi: 10.1186/1745-6215-10-93

60. Jesse DE, Gaynes BN, Feldhousen EB, Newton ER, Bunch S, Hollon SD. Performance of a culturally tailored cognitive-behavioral intervention integrated in a public health setting to reduce risk of antepartum depression: A randomized

controlled trial. Journal of Midwifery & Women's Health. 2015; 60(5), 578-592. doi: 10.1111/jmwh.12308

61. Joiner TE, Voelz ZR, Rudd M. For suicidal young adults with comorbid depressive and anxiety disorders, problem-solving treatment may be better than treatment as usual. Professional Psychology: Research and Practice. 2001; 32(3), 278-282. doi: 10.1037/0735-7028.32.3.278

62. Kaaya SF, Blander J, Antelman G, Cyprian F, Emmons KM, Matsumoto K, et al. Randomized controlled trial evaluating the effect of an interactive group counseling intervention for HIV-positive women on prenatal depression and disclosure of HIV status. AIDS Care. 2013; 25(7), 854-862. doi: 10.1080/09540121.2013.763891

63. Karyotaki E, Klein AE, Ciharova M, Bolinski F, Krijnen L, de Koning L, et al. Guided internet-based transdiagnostic individually tailored Cognitive Behavioral Therapy for symptoms of depression and/or anxiety in college students: A randomized controlled trial, Behaviour Research and Therapy. 2022; 150, doi: 10.1016/j.brat.2021.104028.

64. Katon W, Russo J, Frank E, Barrett J, Williams JW, Oxman T, et al. Predictors of nonresponse to treatment in primary care patients with dysthymia. General Hospital Psychiatry. 2002; 24(1), 20-27. doi: 10.1016/S0163-8343%2801%2900171-2

65. Kelleci M, Buzlu S. The effect of development of problem-solving skills on the depressive symptoms in women in a primary care setting in Turkey. International Public Health Journal. 2011; 1, 31-40. doi: 10.14744/phd.2021.92653

66. Kelly BD, Nur UA, Tyrer P, Casey P. Impact of severity of personality disorder on the outcome of depression. European Psychiatry. 2009; 24(5), 322-326. doi: 10.1016/j.eurpsy.2008.12.004

67. Kendrick T, Simons L, Mynors-Wallis L, Gray A, Lathlean J, Pickering R, et al. A trial of problem-solving by community mental health nurses for anxiety, depression and life difficulties among general practice patients. The CPN-GP study. Health Technology Assessment. 2005; 9(37), 1-104. https://eprints.soton.ac.uk/17535/

68. Kennedy E. Improving preschool outcomes by addressing maternal depression in Head Start. 2020. ClinicalTrials.gov Identifier: NCT04092010. Retrieved from: https://clinicaltrials.gov/show/NCT04092010

69. Kennedy P, Duff J, Evans M, Beedie A. Coping effectiveness training reduces depression and anxiety following traumatic spinal cord injuries. The British Journal of Clinical Psychology. 2003; 42(1), 41-52. Doi: 10.1348/014466503762842002

70. Kenter RM, Cuijpers P, Beekman A, van Straten A. Effectiveness of a web-based guided self-help intervention for outpatients with a depressive disorder: Short-term results from a randomized controlled trial. Journal of Medical Internet research. 2016; 18(3), e80. doi: 10.2196/jmir.4861

71. Kim SH, Lee BG. The effects of a maternal nursing competency reinforcement program on nursing students’ problem-soling ability, emotional intelligence, self-directed learning ability, and maternal nursing performance in Korea: a

randomized controlled trial. Korean Journal of Women Health and Nursing. 2021; 27(3), 230-242. doi: 0.4069/kjwhn.2021.09.13

72. Kindt KC, van Zundert R, Engels RC. Evaluation of a Dutch school-based depression prevention program for youths in highrisk neighborhoods: Study protocol of a two-armed randomized controlled trial. BMC Public Health. 2012; 12(212). doi: 10.1186/1471-2458-12-212

73. Kleiboer A, Donker T, Seekles W, van Straten A, Riper H, Cuijpers P. A randomized controlled trial on the role of support in internet-based problem solving therapy for depression and anxiety. Behaviour Research and Therapy. 2015; 72, 63-71. doi: 10.1016/j.brat.2015.06.013

74. Kobeissi L, Araya R, El Kak F, Ghantous Z, Khawaja M, Khoury B,et al.The relaxation exercise and social support trial-resst: Study protocol for a randomized community based trial. BMC Psychiatry. 2011; 11(142). doi: 10.1186/1471-244X-11-142

75. Kraag G, Van Breukelen GJ, Kok G, Hosman C. 'Learn Young, Learn Fair', a stress management program for fifth and sixth graders: Longitudinal results from an experimental study. Journal of Child Psychology and Psychiatry. 2009; 50(9), 1185-1195. doi: 10.1111/j.1469-7610.2009.02088

76. Kunzler AM, Helmreich I, König J, Chmitorz A, Wessa M, Binder H, Lieb K. Psychological interventions to foster resilience in healthcare students. Cochrane Database of Systematic Reviews. 2020; (7). doi: 10.1002/14651858.CD013684

77. La Greca A. Coping with adolescent peer victimization and reducing anxious/depressed symptoms. 2013. ClinicalTrials.gov Identifier: NCT02011438. Retrieved from: https://clinicaltrials.gov/show/NCT02011438
78. Lester KJ, Mathews A. Davison PS, Burgess JL, Yiend J. Modifying cognitive errors promotes cognitive well being: A new approach to bias modification. Journal of Behavior Therapy and Experimental Psychiatry. 2011; 42(3), 298-308. doi: 10.1016/j.jbtep.2011.01.001
79. Lipinski, C. Cognitive mechanisms of change in adolescent depression. 2015 (Doctoral dissertation, St. John's University, New York).
80. Lou Y, Xia W, Cheung AT, Kwan Ho LL, Zhang J, Xie J, et al. Effectiveness of a mobile device-based resilience training program in reducing depressive symptoms and enhancing resilience and quality of life in parents of children with cancer: randomized controlled trial. Journal of Medical Internet Research. 2021; 23(11): e27639. doi: 10.2196/27639
81. Maddux RE, Riso LP, Klein DN, Markowitz JC, Rothbaum BO, Arnow BA, et al. Select comorbid personality disorders and the treatment of chronic depression with nefazodone, targeted psychotherapy, or their combination. Journal of Affective Disorders. 2009; 117(3), 174-179. doi: 10.1016/j.jad.2009.01.010
82. Maljanen T, Paltta P, Harkanen T, Virtala E, Lindfors O, Laaksonen MA, et al. The cost-effectiveness of short-term psychodynamic psychotherapy and solution-focused therapy in the treatment of depressive and anxiety disorder during a one-year follow-up. Journal of Mental Health Policy and Economics. 2012; 15(1), 13-23. doi: 10.4236/ojpsych.2014.43030

83. Margrain TH, Nollett C, Shearn J, Stanford M, Edwards RT, Ryan B, et al. The Depression in Visual Impairment Trial (DEPVIT): Trial design and protocol. BMC Psychiatry. 2012; 12(57). doi: 10.1186/1471-244X-12-57

84. Martinez V, Martinez P, Vohringer PA, Araya R, Rojas G. Computer-assisted cognitive-behavioral therapy for adolescent depression in primary care clinics in Santiago, Chile (YPSA-M): Study protocol for a randomized controlled trial. Trials. 2014; 15(309). doi: 10.1186/1745-6215-15-309

85. McCarty CA, Weisz JR. Effects of psychotherapy for depression in children and adolescents: What we can (and can't) learn from meta-analysis and component profiling. Journal of the American Academy of Child & Adolescent Psychiatry. 2007; 46(7), 879-886. doi: 10.1097/chi.0b013e31805467b3

86. McFarland CP, Primosch M, Maxson CM, Stewart BT. Enhancing memory and imagination improves problem solving among individuals with depression. Memory & Cognition. 2017; 45(6), 932-939. doi: 10.3758/s13421-017-0706-3

87. McGrady A, Brennan J, Lynch D. Effects of wellness programs in family medicine. Applied Psychophysiology and Biofeedback. 2009; 34(2), 121-126. doi: 10.1007/s10484-009-9084-3

88. McNaughton DB, Cowell JM, Fogg L. Efficacy of a Latino mother-child communication intervention in elementary schools. The Journal of School Nursing. 2015; 31(2), 126-134. doi: 10.1177/1059840514526997

89. Meyer B, Berger T, Caspar F, Beevers CG, Andersson G, Weiss M. Effectiveness of a novel integrative online treatment for depression (Deprexis):

Randomized controlled trial. Journal of Medical Internet Research. 2009; 11(2), e15. doi: 10.2196/jmir.1151

90. Milgrom J, Holt C, Holt CJ, Ross J, Ericksen J, Gemmill AW. Feasibility study and pilot randomised trial of an antenatal depression treatment with infant follow-up. Archives of Women's Mental Health. 2015; 18(5), 717-730. doi: 10.1007/s00737-015-0512-5

91. Moncrieft AE, Llabre MM, McCalla JR, Gutt M, Mendez AJ, Gellman MD, et al. Effects of a multicomponent life-style intervention on weight, glycemic control, depressive symptoms, and renal function in low-income, minority patients with type 2 diabetes: Results of the community approach to lifestyle modification for diabetes randomized controlled trial. Psychosomatic Medicine. 2016; 78(7), 851. doi: 10.1097/PSY.0000000000000348

92. Morokuma I, Shimodera S, Fujita H, Hashizume H, Kamimura N, Kawamura A, et al. Psychoeducation for major depressive disorders: A randomised controlled trial. Psychiatry Research. 2013; 210(1), 134-139. doi: 10.1016/j.psychres.2013.05.018

93. Murphy J, Goldsmith CH, Jones W, Oanh PT, Nguyen VC. The effectiveness of a Supported Self-management task-shifting intervention for adult depression in Vietnam communities: study protocol for a randomized controlled trial. Trials. 2017; 18(1), 209. doi: 10.1186/s13063-017-1924-5

94. Mynors-Wallis L, Gath D, Lloyd-thomas A, Tomlinson D. Randomised controlled trial comparing problem solving treatment with amitryptline and placebo for major

depression in primary care. British Medical Journal. 2019; 310(6977), 441-445. https://www.ncbi.nlm.nih.gov/pmc/articles/PMC2548821/pdf/bmj00580-0035.pdf

95. Narad ME, Raj S, Yeates KO, Taylor HG, Kirkwood MW, Stancin T, et al. Randomized controlled trial of an online problem-solving intervention following adolescent traumatic brain injury: Family outcomes. Archives of Physical Medicine and Rehabilitation. 2019; 100(5), 811-820. doi: 10.1016/j.apmr.2019.01.010

96. O'Kearney R, Kang K, Christensen H, Griffiths K. A controlled trial of a school-based Internet program for reducing depressive symptoms in adolescent girls. Depression and Anxiety. 2009; 26(1), 65-72. doi:10.1002/da.20507

97. Olmedo M, del Barrio V, Santed MA. Gender and previous emotion as predictors of changes in depression and anxiety in adolescence. Ansiedad y Estres. 2000; 6(1), 47-60.

98. Opiyo G. Decreasing stunting by reducing maternal depression in Uganda: A cluster randomized controlled trial (CRCT) for improved nutrition outcomes. 2020. ClinicalTrials.gov Identifier: NCT03573713. Retrieved from: https://clinicaltrials.gov/show/NCT03573713

99. Oxman TE, Barrett JE, Sengupta A, Katon W, Williams JW, Frank E, et al. Status of minor depression or dysthymia in primary care following a randomized controlled treatment. General Hospital Psychiatry. 2001; 23(6), 301-310. doi: 10.1016/S0163-8343%2801%2900166-9

100. Oxman TE, Hegel MT, Hull JG, Dietrich AJ. Problem-solving treatment and coping styles in primary care for minor depression. Journal of Consulting and Clinical Psychology. 2008; 76(6), 933-943. doi: 10.1037/a0012617

101. Papola D, Purgato M, Gastaldon C, Bovo C, Ommeren M, Barbui C, et al. Psychological and social interventions for the prevention of mental disorders in people living in low-and middle-income countries affected by humanitarian crises. Cochrane Database of Systematic Reviews. 2020; (9). doi: 10.1002/14651858.CD012417.pub2.

102. Parker AG, Hetrick SE, Jorm AF, Mackinnon AJ, McGorry PD, Yung AR, et al. The effectiveness of simple psychological and physical activity interventions for high prevalence mental health problems in young people: A factorial randomised controlled trial. Journal of Affective Disorders. 2016; 196, 200-209. doi: 10.1016/j.jad.2016.02.043

103. Păsărelu CR, Dobrean A. A video-based transdiagnostic REBT universal prevention program for internalizing problems in adolescents: Study protocol of a cluster randomized controlled trial. BMC psychiatry. 2018; 18(1), 101. doi: 10.1186/s12888-018-1684-0

104. Pratap A, Renn BN, Volponi J, Mooney SD, Gazzaley A, Arean PA, Anguera JA. Using mobile apps to assess and treat depression in Hispanic and Latino populations: Fully remote randomized clinical trial. Journal of Medical Internet Research. 2018; 20(8), e10130. doi: 10.2196/10130

105. Puskar K, Sereika S, Tusaie-Mumford K. Effect of the Teaching Kids to Cope (TKC) program on outcomes of depression and coping among rural adolescents.

Journal of Child and Adolescent Psychiatric Nursing. 2003; 16(2), 71-80. doi: 10.1111/j.1744-6171.2003.tb00350.x

106. Ranney ML, Goldstick J, Eisman A, Carter PM, Walton M, Cunningham, RM. Effects of a brief ED-based alcohol and violence intervention on depressive symptoms. General Hospital Psychiatry. 2017; 46, 44-48. doi: 10.1016/j.genhosppsych.2017.01.008

107. Richardson ED. Adventure-based therapy and self-efficacy theory: Test of a treatment model for late adolescents with depressive symptomatology. Dissertation Abstracts International: Section B: The Sciences and Engineering. 2003; 63(9-B), 4384. Retrieved from: https://vtechworks.lib.vt.edu/bitstream/handle/10919/26812/Text.pdf?sequence=2

108. Robinson J, Hetrick S, Cox G, Bendall S, Yung A, Yuen HP, et al. The development of a randomised controlled trial testing the effects of an online intervention among school students at risk of suicide. BMC psychiatry. 2014; 14(1), 1-9. doi: 10.1186/1471-244X-14-155

109. Robinson JR, Drotar D, Boutry M. Problem-solving abilities among mothers of infants with failure to thrive. Journal of Pediatric Psychology. 2001; 26(1), 21-32. doi: 10.1093/jpepsy/26.1.21

110. Sampogna G, Fiorillo A, Luciano M, Del Vecchio V, Steardo L, Pocai B, et al. A randomized controlled trial on the efficacy of a psychosocial behavioral intervention to improve the lifestyle of patients with severe mental disorders: Study protocol. Frontiers in Psychiatry. 2018; 9(235). doi: 10.1192/j.eurpsy.2021.2253

111. Sayal K, Roe J, Ball H, Atha C, Kaylor-Hughes C, Guo B, et al. Feasibility of a randomised controlled trial of remotely delivered problem-solving cognitive behaviour therapy versus usual care for young people with depression and repeat self-harm: lessons learnt (e-DASH). BMC Psychiatry. 2019; 19(1), 42. doi: 10.1186/s12888-018-2005-3

112. Schmaling KB, Dimidjian S, Katon W, Sullivan M. Response styles among patients with minor depression and dysthymia in primary care. Journal of Abnormal Psychology. 2002; 111(2), 350-356. doi: 10.1037/0021-843X.111.2.350

113. Seekles W, van Straten A, Beekman A, van Marwijk H, Cuijpers P. Stepped care for depression and anxiety: from primary care to specialized mental health care: A randomised controlled trial testing the effectiveness of a stepped care program among primary care patients with mood or anxiety disorders. BMC Health Services Research. 2009; 9(90). doi: 10.1186/1472-6963-9-90

114. Seekles W, van Straten A, Beekman A, van Marwijk H, Cuijpers P. Effectiveness of guided self-help for depression and anxiety disorders in primary care: A pragmatic randomized controlled trial. Psychiatry Research. 2011; 187(1-2), 113-120. doi: 10.1016/j.psychres.2010.11.015

115. Shimazu K, Shimodera S, Mino Y, Nishida A, Kamimura N, Sawada K, et al. Family psychoeducation for major depression: Randomised controlled trial. The British Journal of Psychiatry. 2011; 198(5), 385-390. doi: 10.1192/bjp.bp.110.078626

116. Shimodera S, Furukawa TA, Mino Y, Shimazu K, Nishida A, Inoue S. Cost-effectiveness of family psychoeducation to prevent relapse in major depression: Results from a randomized controlled trial. BMC Psychiatry. 2012; 12(40). doi: 10.1186/1471-244X-12-40

117. Silverstein M. Problem solving education and neonatal intensive care unit (NICU) mothers (Project Solve). 2016. ClinicalTrials.gov Identifier: NCT01214967. Retrieved from: https://clinicaltrials.gov/ct2/show/NCT01214967

118. Silverstein M, Cabral H, Hegel M, Diaz-Linhart Y, Beardslee W, Kistin CJ, Feinberg E. Problem-solving education to prevent depression among low-income mothers: A path mediation analysis in a randomized clinical trial. JAMA Network Open. 2018; 1(2), e180334. doi: 10.1001/jamanetworkopen.2018.0334

119. Silverstein M, Diaz-Linhart Y, Cabral H, Beardslee W, Broder-Fingert S, Kistin CJ, et al.Engaging mothers with depressive symptoms in care: Results of a randomized controlled trial in head start. Psychiatric Services. 2018; 69(11), 1175-1180. doi: 10.1176/appi.ps.201800173

120. Silverstein M, Diaz-Linhart Y, Cabral H, Beardslee W, Hegel M, Haile W, et al. Efficacy of a maternal depression prevention strategy in head start: A randomized clinical trial. JAMA Psychiatry. 2017; 74(8), 781-789. doi: 10.1001/jamapsychiatry.2017.1001

121. Silverstein M, Feinberg E, Cabral H, Linhart YD, Sandler J, Hegel M, et al. Potential impact of trauma on the ability to prevent depression among low-income mothers. Depression and Anxiety. 2011; 28(6), 478-484. doi: 10.1002/da.20817

122. Silverstein M, Feinberg E, Cabral H, Sauder S, Egbert L, Schainker E, et al. Problem-solving education to prevent depression among low-income mothers of preterm infants: A randomized controlled pilot trial. Archives of Women's Mental Health. 2011; 14(4), 317-324. doi: 10.1007/s00737-011-0222-6

123. Simons CJ, Hartmann JA, Kramer I, Menne-Lothmann C, Hohn P, van Bemmel AL, et al. Effects of momentary self-monitoring on empowerment in a randomized controlled trial in patients with depression. European Psychiatry. 2015; 30(8), 900-906. doi: 10.1016/j.eurpsy.2015.09.004

124. Simons J, Reynolds J, Morison L. Randomised controlled trial of training health visitors to identify and help couples with relationship problems following a birth. British Journal of General Practice. 2001; 51(471), 793-799. https://www.ncbi.nlm.nih.gov/pmc/articles/PMC1314123/

125. Singh N, Minaie MG, Skvarc DR, Toumbourou JW. Impact of a secondary school depression prevention curriculum on adolescent social-emotional skills: Evaluation of the Resilient Families program. Journal of Youth and Adolescence. 2019; 48(6), 1100-1115. doi: 10.1007/s10964-019-00992-6

126. Singh MK, Nimarko AF, Garrett AS, Gorelik AJ, Roybal DJ, Walshaw PD, et al. Changes in intrinsic brain connectivity in family-focused therapy versus standard psychoeducation among youths at high risk for bipolar disorder. Journal of the American Academy of Child & Adolescent Psychiatry. 2021; 60(4), 458-469. doi: 10.1016/j.jaac.2020.07.892

127. Singhal M, Manjula M, Sagar K. Development of a school-based program for adolescents at-risk for depression in India: Results from a pilot study. Asian Journal of Psychiatry. 2014; 10, 56-61. doi: 10.1016/j.ajp.2014.03.011

128. Singhal M, Munivenkatappa M, Kommu JVS, Philip M. Efficacy of an indicated intervention program for Indian adolescents with subclinical depression. Asian Journal of Psychiatry. 2018; 33, 99-104. doi: 10.1016/j.ajp.2018.03.007

129. Singla DR, Kumbakumba E, Aboud FE. Effects of a parenting intervention to address maternal psychological wellbeing and child development and growth in rural Uganda: a community-based, cluster-randomised trial. The Lancet Global Health. 2015; 3(8), e458-e469. doi: 10.1016/S2214-109X(15)00099-6

130. Slee N, Garnefski N, van der Leeden R, Arensman E, Spinhoven P. Cognitive-behavioural intervention for self-harm: randomised controlled trial. The British Journal of Psychiatry. 2008; 192(3), 202-211. doi: 10.1192/bjp.bp.107.037564

131. Smith CE, Curtas S, Kleinbeck SV, Werkowitch M, Mosier M, Seidner DL, et al. Clinical trial of interactive and videotaped educational interventions reduce infection, reactive depression, and rehospitalizations for sepsis in patients on home parenteral nutrition. Journal of Parenteral and Enteral Nutrition. 2003; 27(2), 137-145. doi: 10.1177/0148607103027002137

132. Spence SH, Sheffield JK, Donovan CL. Preventing adolescent depression: An evaluation of the Problem Solving For Life program. Journal of Consulting and Clinical Psychology. 2003; 71(1), 3-13. doi: 10.1037/0022-006X.71.1.3

133. Spence SH, Sheffield JK, Donovan CL. Long-term outcome of a school-based, universal approach to prevention of depression in adolescents. Journal of

Consulting and Clinical Psychology. 2005; 73(1), 160-167. doi:10.1037/0022-006X.73.1.160

134. Stavros HJ. Reducing the effects of maternal depression on the family: A study of the effectiveness of a short-term preventive intervention program. Dissertation Abstracts International Section A: Humanities and Social Sciences, 63(1-A). 2002; 365. Retrieved from: https://www.proquest.com/dissertations-theses/reducing-effects-maternal-depression-on-family/docview/304722489/se-2

135. Steinhardt M, Dolbier C. Evaluation of a resilience intervention to enhance coping strategies and protective factors and decrease symptomatology. Journal of American College Health. 2008; 56(4), 445-453. doi: 10.1.1.470.5701

136. Stewart CD, Quinn A, Plever S, Emmerson B. Comparing cognitive behavior therapy, problem solving therapy, and treatment as usual in a high risk population. Suicide and Life-Threatening Behavior. 2009; 39(5), 538–547. doi: 10.1521/suli.2009.39.5.538

137. Syrjala KL, Yi JC, Artherholt SB, Romano JM, Crouch ML, Fiscalini AS, et al. An online randomized controlled trial, with or without problem-solving treatment, for long-term cancer survivors after hematopoietic cell transplantation. Journal of Cancer Survivorship. 2018; 12(4), 560-570. doi: 10.1007/s11764-018-0693-9

138. Tak YR, Van Zundert RM, Kuijpers RC, Van Vlokhoven BS, Rensink HF, Engels RC. A randomized controlled trial testing the effectiveness of a universal school-based depression prevention program 'Op Volle Kracht' in the Netherlands. BMC Public Health. 2012; 12(1), 1-9. doi: 10.1186/1471-2458-12-21

139. Thompson EA, Eggert LL, Randell BP, Pike KC. Evaluation of indicated suicide risk prevention approaches for potential high school dropouts. American Journal of Public Health. 2001; 91(5), 742-752. doi: 10.2105/ajph.91.5.742

140. Thornett AM, Mynors-Wallis LM. Credibility of problem-solving therapy and medication for the treatment of depression among primary care patients. Medical Science Monitor. 2002; 8(3), CR193-196. https://pubmed.ncbi.nlm.nih.gov/11887035/

141. Thummathai K, Sethabouppha H, Chanprasit C, Lasuka D. Depression prevention in adolescents based on buddhism and sufficiency economy philosophy. Archives of Psychiatric Nursing. 2020; 34(1), 70-74. doi: 10.1016/j.apnu.2019.10.005

142. University of Nottingham. RCT of the clinical and cost effectiveness of cognitive behaviour therapy (CBT) delivered remotely versus treatment as usual in adolescents and young adults with depression who repeatedly self-harm. 2015. ClinicalTrials.gov Identifier: NCT02377011. Retrieved from: https://clinicaltrials.gov/show/NCT02377011

143. van der Voort TY, van Meijel B, Goossens PJ, Hoogendoorn AW, Draisma S, Beekman A, et al. Collaborative care for patients with bipolar disorder: randomised controlled trial. The British Journal of Psychiatry. 2015; 206(5), 393-400.

144. Van't Hof E, Stein DJ, Marks I, Tomlinson M, Cuijpers P. The effectiveness of problem solving therapy in deprived South African communities: results from a pilot study. BMC Psychiatry. 2011; 11(1), 156. doi: 10.1186/1471-244X-11-156

145. Van Horne BS, Nong YH, Cain CM, Sampson M, Greeley CS, Puryear L. A promising new model of care for postpartum depression: A randomised controlled trial of a brief home visitation program conducted in Houston, Texas, USA. Health Soc Care Community. 2022; 30(5):e2203-e2213. doi: 10.1111/hsc.13658. Epub 2021 Dec 5. PMID: 34866254.

146. van Straten A, Cuijpers P, Smits N. Effectiveness of a web-based self-help intervention for symptoms of depression, anxiety, and stress: Randomized controlled trial. Journal of Medical Internet research. 2008; 10(1), e7. doi:10.2196/jmir.954

147. Vázquez González FL, Otero Otero P, Torres Iglesias A, Hermida García E, Blanco Seoane V, Díaz Fernández O. A brief problem-solving indicated-prevention intervention for prevention of depression in nonprofessional caregivers. Psicothema. 2013; 25(1), 87-92. doi:10.7334/psicothema2012.89

148. Wade SL, Cassedy AE, McNally KA, Kurowski BG, Kirkwood MW, Stancin T, et al. A randomized comparative effectiveness trial of family-problem-solving treatment for adolescent brain injury: Parent outcomes from the Coping with Head Injury through Problem Solving (CHIPS) study. The Journal of Head Trauma Rehabilitation. 2019; 34(6), E1-E9. doi: 10.1097/HTR.0000000000000487

149. Wade SL, Karver CL, Taylor HG, Cassedy A, Stancin T, Kirkwood MW, et al. Counselor-assisted problem solving improves caregiver efficacy following adolescent brain injury. Rehabilitation Psychology. 2014; 59(1), 1-9. doi: 10.1037/a0034911

150. Wade SL, Taylor H, Cassedy A, Zhang N, Kirkwood MW, Brown TM, et al. Long-term behavioral outcomes after a randomized, clinical trial of counselor-assisted problem solving for adolescents with complicated mild-to-severe traumatic brain injury. Journal of Neurotrauma. 2015; 32(13), 967-975. doi: 10.1089/neu.2014.3684

151. Wade SL, Walz NC, Carey J, McMullen KM, Cass J, Mark E, et al. A randomized trial of teen online problem solving: Efficacy in improving caregiver outcomes after brain injury. Health Psychology. 2012; 31(6), 767-776. doi: 10.1037/a0028440

152. Wade SL, Carey J, Wolfe CR. An online family intervention to reduce parental distress following pediatric brain injury. Journal of consulting and clinical psychology. 2006; 74(3), 445. doi: 10.1037/0022-006X.74.3.445

153. Wagner GJ, McBain RK, Akena D, NgoV, Nakigudde J, Nakku J, et al. Maternal depression treatment in HIV (M-DEPTH): Study protocol for a cluster randomized controlled trial. Medicine. 2019; 98(27). doi: 10.1097/MD.0000000000016329

154. Warmerdam L, Smit F, van Straten A, Riper H, Cuijpers P. Cost-utility and cost-effectiveness of internet-based treatment for adults with depressive symptoms: Randomized trial. Journal of medical Internet research. 2010; 12(5), e53. doi: 10.2196/jmir.1436

155. Wong PW, Fu KW, Chan KY, Chan WS, Liu PM, Law YW, et al. Effectiveness of a universal school-based programme for preventing depression in Chinese adolescents: A quasi-experimental pilot study. Journal of Affective Disorders. 2012; 142(1-3), 106-114. doi: 10.1016/j.jad.2012.03.050

156. Wootton AR, Legnitto DA, Gruber VA, Dawson-Rose C, Neilands TB, Johnson MO, et al. Telehealth and texting intervention to improve HIV care engagement, mental health and substance use outcomes in youth living with HIV: a pilot feasibility and acceptability study protocol. BMJ open. 2019; 9(7), e028522. doi: 10.1136/bmjopen-2018-028522

157. Zhang N, Kaizar EE, Narad ME, Kurowski BG, Yeates KO, Taylor H, et al. Examination of injury, host, and social-environmental moderators of online family problem solving treatment efficacy for pediatric traumatic brain injury using an individual participant data meta-analytic approach. Journal of Neurotrauma. 2019; 36(7), 1147-1155. doi: 10.1089/neu.2018.5885

158. Zhang DX, Lewis G, Araya R, Tang WK, Mak WW, Cheung FM, et al. Prevention of anxiety and depression in Chinese: a randomized clinical trial testing the effectiveness of a stepped care program in primary care. Journal of Affective Disorders. 2014; 169, 212-220. doi: 10.1016/j.jad.2014.08.015
