# Supplementary material for: Problem-solving interventions and depression among adolescents and young adults: A systematic review of the effectiveness of problem-solving interventions in preventing or treating depression
Source: PLoS One. 2023 Aug 29;18(8):e0285949. doi: 10.1371/journal.pone.0285949 (PMC10464969; doi:10.1371/journal.pone.0285949)
Supplement: S1 Table — (PDF) [file pone.0285949.s003.pdf]

S1 Table. Individual risk of bias assessments using Cochrane RoB2 Tool by Domain (1-5) and Overall (6)

Study (First author and year)	Domain 1. Randomisation process	Domain 2. Deviations from intended interventions	Domain 3. Missing outcome data	Domain 4. Measurement of outcome	Domain 5. Selection of the reported result	Domain 6. Overall risk of bias
**Bird 2018**	Low risk of bias	Some concerns	Some concerns	Some concerns	Some concerns	Some concerns
**Brugha 2000**	Low risk of bias	Some concerns	Low risk of bias	Some concerns	Some concerns	Some concerns
**Chibanda 2014**	Some concerns	Some concerns	Some concerns	High risk of bias	Some concerns	Some concerns
**Dietz 2014**	Some concerns	Some concerns	Low risk of bias	Low risk of bias	Some concerns	Some concerns
**Eskin 2008**	Some concerns	Some concerns	Some concerns	Some concerns	Some concerns	Some concerns
**Fitzpatrick 2005**	Some concerns	Some concerns	Low risk of bias	Some concerns	Some concerns	Some concerns
**Gaffney 2014**	High risk of bias	Some concerns	Low risk of bias	Some concerns	Some concerns	Some concerns
**Gureje 2019**	Some concerns	Some concerns	Low risk of bias	Low risk of bias	Some concerns	Some concerns
**Haeffel 2017**	Some concerns	High risk of bias	High risk of bias	High risk of bias	Some concerns	High risk of bias
**Hallford 2016**	Low risk of bias	Low risk of bias	Some concerns	Some concerns	Some concerns	Some concerns
**Hoek 2012**	Low risk of bias	Some concerns	Low risk of bias	Some concerns	Some concerns	Some concerns
**Hood 2018**	Some concerns	Some concerns	Low risk of bias	Some concerns	Some concerns	Some concerns

**Houston 2017**	Some concerns	Some concerns	Low risk of bias	Some concerns	Some concerns	Some concerns
**Kolko 2000**	Some concerns	Some concerns	Low risk of bias	High risk of bias	Some concerns	Some concerns
**Makover 2019**	Some concerns	Some concerns	Low risk of bias	Some concerns	Some concerns	Some concerns
**Malik 2021**	Low risk of bias	Low risk of bias	Low risk of bias	Low risk of bias	Low risk of bias	Low risk of bias
**Michelson 2020**	Low risk of bias	Low risk of bias	Low risk of bias	Low risk of bias	Low risk of bias	Low risk of bias
**Miklowitz 2014**	Some concerns	Some concerns	Low risk of bias	Low risk of bias	Some concerns	Some concerns
**Miklowitz 2020**	Low risk of bias	Low risk of bias	Low risk of bias	Low risk of bias	Low risk of bias	Low risk of bias
**Parker 2016**	Low risk of bias	Some concerns	Some concerns	Low risk of bias	Some concerns	Some concerns
**Psaros 2022**	Low risk of bias	Low risk of bias	Low risk of bias	Some concerns	Low risk of bias	Low risk of bias
**Weissberg -Benchell 2020**	Some concerns	Some concerns	Low risk of bias	Low risk of bias	Some concerns	Some concerns
**Xavier 2019**	Low risk of bias	Low risk of bias	Low risk of bias	Low risk of bias	Low risk of bias	Low risk of bias
